# Visceral-to-subcutaneous fat ratio is a time-stable predictor of survival and toxicity in advanced hepatocellular carcinoma

**DOI:** 10.1007/s00432-026-06570-1

**Published:** 2026-08-05

**Authors:** Salvatore Corallo, Margherita Rimini, Carlo Paolo Scolari, Catherine Klersy, Elisa Mattavelli, Chandra Bortolotto, Salvatore Dierna, Giulia Gambini, Amanda Casirati, Francesco Agustoni, Angioletta Lasagna, Silvia Chiellino, Silvia Foti, Federico Rossari, Silvia Camera, Francesco Serra, Anna Pagani, Sofia Marino, Lorenzo Preda, Riccardo Caccialanza, Paolo Pedrazzoli, Emanuele Cereda, Andrea Casadei-Gardini

**Affiliations:** 1 Department of Internal Medicine and Medical Therapy, University of Pavia, Corso Strada Nuova 65, 27100 Pavia, Italy; 2 Department of Oncology, Comprehensive Cancer Center, Fondazione IRCCS Policlinico San Matteo, Pavia, Italy; 3 Department of Oncology, IRCCS San Raffaele Hospital, Milan, Italy; 4 Department of Oncology, Vita-Salute San Raffaele University, Milan, Italy; 5 BERD Unit, Comprehensive Cancer Center, Fondazione IRCCS Policlinico San Matteo, Pavia, Italy; 6 Clinical Nutrition and Dietetics Unit, Comprehensive Cancer Center, Fondazione IRCCS Policlinico San Matteo, Pavia, Italy; 7 Department of Clinical, Surgical, Diagnostic and Pediatric Sciences, University of Pavia, Pavia, Italy; 8 Diagnostic Imaging and Radiotherapy Unit, Comprehensive Cancer Center, Fondazione IRCCS Policlinico San Matteo, Pavia, Italy; 9 Pediatric Endocrinology Unit, Department of Pediatrics, IRCCS Istituto Giannina Gaslini, Genoa, Italy; 10 Department of Neuroscience, Rehabilitation, Ophthalmology, Genetics, Maternal and Child Health, University of Genoa, Genoa, Italy; 11 Department of Oncology and Hematology-Oncology, University of Milan, Milan, Italy

**Keywords:** VAT-SAT ratio, Adipose tissue, Dose-limiting toxicities, Hepatocellular carcinoma

## Abstract

**Purpose:**

Nutritional status significantly impacts the prognosis of patients with hepatocellular carcinoma (HCC). Computed tomography (CT) has become a key tool for assessing body composition (BC), with abnormal patterns increasingly linked to reduced survival. However, BC is dynamic and may change during treatment, potentially affecting treatment outcomes. This study explores how BC affects survival and dose-limiting toxicities (DLTs) in patients with advanced HCC receiving first-line treatment, as well as changes in BC over the course of treatment.

**Methods:**

This retrospective study included patients with advanced HCC who received first-line systemic therapy. The Lumbar skeletal muscle index and adipose tissue indices were measured via CT analysis at baseline and at the best response. Cox regression and generalized linear models were used for analysis.

**Results:**

The study included 113 patients treated with tyrosine kinase inhibitors (70%) or immune checkpoint inhibitors (30%), primarily atezolizumab and bevacizumab. Univariable analyses identified a high visceral-to-subcutaneous adipose tissue ratio (VSR) and muscle depletion as predictors of poor survival. The association between elevated VSR and reduced survival was further confirmed in bivariable models adjusted for relevant prognostic factors. Elevated VSR was also associated with an increased incidence of DLT (hazard ratio [HR]: 1.16, 95% CI: 1.08–1.26, *p* < 0.001). Although most patients experienced muscle and fat loss during treatment, VSR remained stable. At the time of best radiologic response, high VSR remained associated with poorer survival outcomes (HRs for progression-free survival and overall survival: 1.37 and 1.12, respectively; *p* < 0.001).

**Conclusions:**

Although validation in larger studies is needed, our study suggests that VSR is a robust, time-stable predictor of survival and toxicity in patients with advanced HCC.

**Supplementary Information:**

The online version contains supplementary material available at https://doi.org/10.1007/s00432-026-06570-1.

## Introduction

Hepatocellular carcinoma (HCC) is one of the most common cancers worldwide and the leading cause of mortality among patients with cirrhosis (Akinyemiju et al. 2017; Sung et al. 2021). Despite advancements in screening and surveillance, over two-thirds of patients are diagnosed with advanced disease(Forner et al. 2012; Park et al. 2015).

Systemic therapy for advanced hepatocellular carcinoma has evolved markedly over the past decade. Sorafenib, introduced in 2007, was the first agent to improve overall survival in this setting, establishing a reference standard through multikinase inhibition of proliferative and angiogenic pathways (Llovet et al. 2008).

Lenvatinib later emerged as a mechanistically distinct alternative tyrosine kinase inhibitor (TKI), with the REFLECT trial demonstrating non-inferiority to sorafenib and confirming that antiangiogenic signaling could be targeted through distinct inhibitory profiles (Kudo et al. 2018).

A transformative shift occurred with atezolizumab plus bevacizumab, which significantly improved survival compared with sorafenib and established immunotherapy-based combinations as a first-line standard of care. This strategy builds on synergistic immune activation and vascular modulation (Finn et al. 2020).

Concurrently, the STRIDE regimen (tremelimumab plus durvalumab) introduced a dual immune-checkpoint inhibitor (ICI) strategy that demonstrated a survival benefit versus sorafenib, thereby expanding the immunotherapy landscape through a mechanism distinct from vascular endothelial growth factor-mediated approaches (Abou-Alfa et al. 2022). Similarly, the combination of ipilimumab and nivolumab has further supported the role of dual immune checkpoint blockade in HCC, with evidence demonstrating clinical activity and reinforcing the biological rationale for intensified immunomodulation (Yau et al. 2025). Several systems have been proposed to classify patients with hepatocellular carcinoma, with the Barcelona Clinic Liver Cancer (BCLC) staging system serving as the most widely adopted worldwide for treatment stratification and clinical decision-making (Reig et al. 2022). However, the BCLC framework does not account for nutritional status when stratifying patient risk and guiding treatment choices. Nutritional status represents a key dimension of patient fitness, especially given the liver’s central role in energy and nutrient metabolism and the frequent development of hypermetabolic, catabolic states in chronic liver disease (Cabré and Gassull 1993). Protein-energy malnutrition is commonly observed in patients with liver cirrhosis and is linked to poor prognosis and increased complications (Huisman et al. 2011; Belarmino et al. 2019). Recent findings have also highlighted the important role of adipose tissue distribution in the metabolism and outcomes of patients with HCC (Ebadi et al. 2017). Adipose tissue includes subcutaneous adipose tissue (SAT), visceral adipose tissue (VAT), and intramuscular adipose tissue (IMAT), each with distinct metabolic and endocrine functions (Bouchard et al. 1993). Emerging evidence suggests that both adipose tissue quantity and quality (radiodensity) may significantly influence outcomes in patients with cirrhosis and HCC (von Hessen et al. 2021; Shi et al. 2023). Adipose tissue plays an active role in regulating endocrine function, angiogenesis, and immune responses, which are pathways directly targeted by current treatment strategies for advanced HCC (Herold and Kalucka 2021; Blaszczak et al. 2021). This interaction may contribute to the so-called “obesity paradox,” wherein obesity, typically associated with poorer outcomes, is paradoxically linked to improved outcomes in patients receiving immune checkpoint inhibitors (Alden et al. 2025; Chen et al. 2025).

Body composition is not static but highly dynamic in patients with cancer and may change during treatment. Recent evidence suggests that skeletal muscle depletion and unfavorable adipose tissue distribution patterns predict poorer prognosis in HCC patients, particularly in advanced stages and during treatment with TKIs (Imai et al. 2015, 2020, 2024). However, limited data exist regarding how body composition dynamics affect treatment strategies for advanced HCC.

This study aims to investigate the impact of body composition parameters on survival and the incidence of dose-limiting toxicities (DLTs) in patients with HCC receiving first-line systemic treatment. Additionally, it seeks to investigate changes in body composition during treatment and their prognostic implications.

## Materials and methods

### Study population and design

We designed a retrospective observational study using prospectively collected routine institutional data. The study cohort included patients with HCC from two Centers in Italy: Fondazione IRCCS Policlinico San Matteo in Pavia, and IRCCS San Raffaele Scientific Institute Hospital in Milan. Potentially eligible patients were selected from a prospectively established database of the Centers between January 2018 and January 2024.

Inclusion criteria were: (1) adult age (18 years or older); (2) histologically or clinically confirmed diagnosis of HCC, according to the European Association for the Study of the Liver (EASL) clinical practice guidelines (Sangro et al. 2025); (3) advanced disease classified as BCLC stage C, or B (Reig et al. 2022), but unfit for or having failed locoregional therapies; (4) no prior systemic treatment for HCC; (5) receipt of first-line therapy for HCC; (6) availability of abdominal CT imaging before the initiation of first-line treatment (baseline [within eight weeks]) for central review.

The choice of systemic treatment regimen was determined either by the treating physician’s clinical judgment or by the availability and approval status of specific agents at the time of treatment initiation, which varied across the study period.

The study protocol was approved by the local ethics committee (“Comitato Etico Territoriale Lombardia 6”, protocol ID: 0039939/23), and the analyses were conducted in accordance with the current version of the Declaration of Helsinki. The review board waived the requirement for study-specific signed informed consent due to the study’s retrospective nature. Data were reported in agreement with the STROBE checklist.

### Clinical data collection

Clinical data were obtained from the medical charts in both cohorts. The following information was recorded: patient demographics and anthropometrics (weight and height), clinical history, the etiology of underlying liver disease -including alcohol-related liver disease (ALD), chronic hepatitis B virus (HBV), chronic hepatitis C virus (HCV) infection, metabolic dysfunction-associated steatotic liver disease/metabolic-dysfunction associated steatohepatitis (MASLD/MASH), and metabolic and alcohol-related liver disease (MetALD) (European Association for the Study of the Liver et al. 2024), Child–Pugh score, comorbidities, tumor characteristics, BCLC classification (Reig et al. 2022), laboratory results, anticancer treatments. The RedCap platform (RedCap 15.5.13—© 2025 Vanderbilt University) at the coordinating Center was used for data collection.

### Body composition parameters and CT analysis

Body mass index (BMI) was calculated from measured weight and height using the formula: weight/height^2 (in kg/m^2). Skeletal muscle (SM), VAT, SAT, and IMAT were quantified by analyzing unenhanced cross-sectional CT scan images performed according to clinical practice at two time points: within 8 weeks before the start of first-line treatment (baseline – in all patients), and at the time of the best radiologic response (if available). Radiologic responses were retrospectively assessed by the treating physicians, without formal RECIST assessment. CT examinations were performed on different scanners. Image parameters were as follows: 120 kVp, automatic mAs modulation, slice thickness < 2 mm, soft-tissue kernel, in accordance with clinical practice. Two radiologists with certified experience in oncologic imaging extracted a single axial CT image of the abdomen at the level of the third lumbar vertebra (L3) for each patient at each time point, as previously reported (Shen et al. 2004). Vertebrae were identified numbering them from the basicranium. Images were selected by consensus as those at the center of the vertebral soma; if artifacts, severe scoliosis, or ambiguous vertebral numbering were present, the patient was excluded. These cross-sectional CT images were then analyzed by two trained investigators (blinded to all clinical and laboratory data) to determine SM and abdominal adipose tissue parameters using Slice-O-Matic software (version 5.0; Tomovision, Montreal, Canada). The inter-observer reproducibility was excellent for all parameters, ranging from 0.983 to 1 (Supplementary Fig. 1).

Skeletal and adipose tissues were identified using pre-established Hounsfield unit (HU) thresholds (radiodensity) as follows: − 29 to + 150 HU for SM, − 190 to − 30 HU for SAT (Mitsiopoulos et al. 1998), − 150 to − 50 HU for VAT (Miller et al. 1998; Mitsiopoulos et al. 1998), and − 190 to − 30 HU for IMAT (Mitsiopoulos et al. 1998). Muscle cross-sectional areas included the psoas, erector spinae, quadratus lumborum, transversus abdominis, external and internal obliques, and rectus abdominis muscles that were segmented separately. The SAT was estimated as the area between the skin line and the outer abdominal wall, and this estimate was subtracted from the total abdominal fat mass measured within the abdominal cavity to yield the VAT. Skeletal muscle area (SMA, cm2) and adipose tissue areas at the L3 were first automatically quantified in cm2 by summing tissue pixels and multiplying by the pixel surface area (a representative image used for analysis is shown in Supplementary Fig. 2).

As previously described (Fujiwara et al. 2015), these outputs were then normalized for the squared height in meters (m2) and reported as the SM Index (SMI), SAT Index (SATI), VAT Index (VATI), and IMAT Index (IMATI). Adipose tissue distribution was explored by calculating the VAT/SAT ratio (VSR).

In this study, muscle depletion was defined using sex- and body mass index-specific (BMI) cut-off points as established by Martin et al.: SMI < 41 cm2/m2 for female patients; SMI < 43 cm2/m2 for males with a BMI < 25 kg/m2 and SMI < 53 cm2/m2 for males with a BMI ≥ 25 kg/m2 (Martin et al. 2013). Since no validated cut-offs exist for SATI, VATI, IMATI, VSR, SAT, and VAT density, patients were classified using the median values of the study cohort, as previously reported (von Hessen et al. 2021).

### Study endpoints and outcome ascertainment

The primary objective was to examine the relationship between baseline BC parameters and survival rates. The safety endpoint was the incidence of DLTs during first-line treatment. Secondary objectives were progression-free survival, assessment of changes in body composition during treatment, and the association between body composition and clinical variables.

All-cause mortality was ascertained using the following methods: linkage to municipal registries, in-office visits, inquiries by mail or telephone to participants or proxy respondents. Overall survival (OS) was defined as the time from the first day of first-line treatment to death or last contact (censored up to January 2025). Progression-free survival (PFS) was defined as the time from the first day of first-line treatment to disease progression or death from any cause. DLT was defined as any severe toxicity that led to treatment delay, reduction, or discontinuation. Discontinuation secondary to disease progression was not accounted for in the DLT definition.

### Statistical analysis

Analyses were performed using the Stata software (release 19.5, StataCorp, College Station, TX, USA). All A 2-sided *p*-value < 0.05 was considered statistically significant. Continuous variables were presented as either mean and standard deviation or as median and interquartile range (IQR), depending on the distribution. Categorical variables were displayed as counts and percentages. For the purpose of the analysis, continuous variables were dichotomized at their median value. Comparisons between study groups (e.g., center and sex) were conducted using Student’s t-test or the Mann–Whitney U-test for continuous variables and Fisher’s exact test for categorical variables. The Pearson correlation coefficient (r) was computed to measure the association of nutritional variables and BMI. The effect of each of the body composition variables (SMI, VATI, SATI, IMATI, VSR, VAT, and SAT density) on survival was analysed by categorizing the data into two categories for each variable: muscle depleted or not, according to Martin et al. (Martin et al. 2013), and high (above the median) and low (below the median) for the other BC parameters. For the time-to-event endpoints, we computed the median (event-free) survival, according to the Kaplan–Meier method, and derived HR and 95% confidence intervals (95%CI) from a Cox model. We fitted both univariable and bivariable models including VSR and the clinical variables in turn. Their interaction was also assessed; if significant, subgroup analysis by clinical features was performed. Landmark analysis at the time of the best response was used to assess the prognostic role of changes in body composition. Given the distribution of the outcomes and the high risk of overfitting, no multivariable models could be performed. Generalized linear regression models for repeated measures were fitted to assess changes in body composition. Huber-White robust standard errors were computed to account for intra-patient correlation. Bivariate models including treatment were also fitted, and their interaction was assessed. In no case interaction was elicited.

## Results

A total of 113 patients were included in the study (Supplementary Fig. 3). Baseline clinical and laboratory characteristics are presented in Table 1. Most participants were male (87.6%), and the median age was 72 years (IQR: 66–78). Nearly half of the patients (44.2%) had extrahepatic metastases. Most patients (70%) were treated with TKIs, specifically lenvatinib or sorafenib (41.6% and 28.3%, respectively). Additionally, 34 patients (30.0%) received ICIs. Baseline characteristics did not differ significantly between the two recruiting centers, except for a higher proportion of patients with extrahepatic metastases (58.1% vs 27.5%, *p* = 0.001) and Eastern Cooperative Oncology Group (ECOG) Performance Status of 1 (56.5% vs 23.5%, *p* = 0.001) in the San Matteo cohort.

**Table 1:** Patients’ characteristics

Characteristics	Overall N = 113 (%)	San Raffaele cohort N = 51 (%)	San Matteo cohort N = 62 (%)	*p*-value
*Age, years,*				
Median (IQR)	72 (66 to 78)	72.5 (67.5 to 78)	72 (62 to 79)	0.078
< 70/ ≥ 70 years	45/68 (39.8/60.2)	19/32 (37.3/62.7)	26/36 (41.9/58.1)	0.571
*Sex*				
Male/female	99/14 (87.6/12.4)	45/6 (88.2/11.8)	54/8 (87.1/12.9)	0.855
*ECOG PS*				0.001*
0	65 (57.5)	38 (74.5)	27 (43.5)	
1	47 (41.6)	12 (23.5)	35 (56.5)	
2	1 (0.9)	1 (2.0)	0	
*Etiology*				
HBV	9 (8.0)	1 (2.0)	8 (12.9)	0.064
HCV	43 (38.1)	18 (35.3)	25 (40.3)	0.584
HBV and HCV	6 (5.3)	1 (2.0)	5 (8.1)	0.150
ALD	10 (8.8)	2 (3.9)	8 (12.9)	0.094
MetALD	11 (9.7)	9 (17.6)	2 (3.2)	0.026*
Mixed (ALD plus viral)	8 (7.1)	2 (3.9)	6 (9.7)	0.235
MASLD/MASH	23 (20.4)	15 (29.4)	8 (12.9)	0.030*
UNK	3 (2.6)	3 (5.9)	0	0.150
*Comorbidities*				
Diabetes	36 (31.9)	15 (29.4)	21 (33.9)	0.613
Ischemic cardiomyopathy	13 (11.5)	9 (17.6)	4 (6.5)	0.063
Atrial fibrillation	12 (10.6)	6 (11.7)	6 (9.7)	0.720
*Child–Pugh class*				
A/B	91/22 (80.5/19.5)	41/10 (80.4/19.6)	50/12 (80.6/19.4)	0.973
*Macrovascular invasion*				0.493
Yes/No	15/98 (13.3/86.7)	8 (15.7/84.3)	7/55 (11.3/88.7)	0.493
*Extrahepatic metastasis*				
Yes/No	50/63 (44.2/55.8)	14/37 (27.5/72.5)	36/26 (58.1/41.9)	0.001*
*BCLC stage*				
B/C	53/60 (46.9/53.1)	29/22 (56.9/43.1)	24/38 (38.7/61.3)	0.054
*ALBI Grade*				
1/2/3/UNK	36/60/6/11 (31.9/53.1/5.3/9.7)	14/24/2/11 (27.5/47.1/3.9/21.6)	22/36/4/0 (35.5/58.1/6.5/0)	0.002*
*AFP (U/ml)†*				
< 400/ ≥ 400 ng/ml/	74/28 (65.5/24.8)	27/17 (52.9/33.3)	47/11 (75.8/17.7)	0.043*
*Laboratory parameters at baseline*				
Albumin (g/l), mean ± SD	36.4 ± 5.30	36.5 ± 5.1	36.4 ± 5.4	0.989
NLR, mean ± SD	3.53 ± 2.98	3.56 ± 3.44	3.51 ± 2.63	0.794
*First-line treatment regimen*				
Sorafenib	32 (28.3)	4 (7.8)	28 (45.2)	< 0.001*
Lenvatinib	47 (41.6)	30 (58.8)	17 (27.4)	
Atezolizumab-bevacizumab	17 (15.0)	3 (5.9	14 (22.6)	
Other ICI-based regimens	17 (15.0)	14 (27.5)	3 (4.8)	
*Best response to first-line treatment*				
CR	5 (4.4)	4 (7.8)	1 (1.6)	0.057
PR	28 (24.8)	13 (25.5)	15 (24.2)	
SD	38 (33.6)	14 (27.5)	24 (38.7)	
PD	31 (27.4)	13 (25.5)	18 (29.0)	
UNK	11 (9.7)	7 (13.7)	4 (6.5)	

ALBI grade, albumin-bilirubin grade; AFP, alpha-fetoprotein; ALD, alcohol-related liver disease; ALT, alanine aminotransferase; AST, aspartate aminotransferase; BCLC, Barcelona Clinic Liver Cancer; ECOG PS, Eastern Cooperative Oncology Group Performance Status; HBV, hepatitis B virus; HCV, hepatitis C virus; ICI, immune checkpoint inhibitors; IQR, interquartile range; MASLD/MASH, metabolic dysfunction-associated steatotic liver disease or metabolic dysfunction-associated steatohepatitis; MetALD, metabolic and alcohol-related liver disease; NA, not applicable; UNK, unknown.

†:Percentages may not total 100 due to missing values. *: Statistically significant.

Baseline BC parameters are summarized in Supplementary Table 1. Most patients were of normal weight, and muscle depletion (Martin et al. 2013) was observed in 41.6% of patients. SAT, VAT, and SMA were moderately correlated with BMI (r = 0.73, r = 0.61, and r = 0.47, respectively), while IMAT and VSR showed no correlations with BMI (Supplementary Fig. 4). As previously specified, patients were divided into low and high groups for SATI, VATI, IMATI, VSR, VAT, and SAT density based on the median BC values observed in our cohort (selected cut-off for SATI, VATI, IMATI, VSR, SAT density, and VAT density were 48, 50, 5, 1, − 95, and − 88, respectively).

After a median follow-up of 34.7 months, 101 patients (89.4%) progressed to first-line treatment, and 89 patients (78.8%) died. Median PFS for first-line treatment was 7.62 months (IQR: 4.08–14.64); median OS was 14.03 months (IQR: 7.86–26.25). In univariable Cox regression analysis, age ≥ 70 years, alpha-fetoprotein (AFP) > 400 ng/ml, albumin-bilirubin (ALBI) grade > 1, Child–Pugh B, and evidence of macrovascular invasion were associated with an increased risk of progression to first-line treatment and mortality. In contrast, therapy with ICIs was associated with improved PFS and OS compared to TKIs (HR: 0.58 and 0.62, respectively) (Table 2).

**Table 2:** Progression-free survival and overall survival by univariable Cox model

Characteristic	Univariable model for PFS			Univariable model for OS		
	HR	95% CI	*p* value	HR	95% CI	*p* value
Gender (female/male)	1.28	0.89–1.85	0.189	1.18	0.75–1.86	0.470
Age (≥ 70/ < 70 years)	1.18	1.06–1.31	0.003*	1.53	1.45–1.61	< 0.001*
AFP (> 400/ ≤ 400 ng/ml)	1.46	1.43–1.49	< 0.001*	1.57	1.43–1.72	< 0.001*
Albi Grade (≥ 2/1)	1.80	1.54–2.09	< 0.001*	2.47	2.00–3.07	< 0.001*
Child (B/A)	1.91	1.27–2.86	0.002*	2.66	1.14–6.21	0.023*
BCLC (C/B)	1.07	0.91–1.26	0.414	1.11	0.92–1.32	0.275
Macrovascular invasion (yes/no)	3.16	1.70–5.86	< 0.001*	2.60	1.24–5.44	0.011*
Etiology (MASLD/Others)	1.23	0.88–1.71	0.233	1.19	0.97–1.47	0.100
Treatment regimen (Immunotherapy/TKI)	0.58	0.40–0.83	0.003*	0.62	0.44–0.87	0.006*
Diabetes (yes/no)	1.39	0.83–2.31	0.206	1.63	0.92–2.88	0.092
Body composition variables						
VATI (high/low)	1.26	1.04–1.54	0.021*	1.12	0.70–1.81	0.638
SATI (high/low)	1.13	0.7–1.64	0.508	1.01	0.44–2.33	0.973
IMATI (high/low)	1.45	0.85–2.48	0.170	1.27	0.76–2.10	0.363
Muscle depletion (yes/no)	1.29	1.03–1.61	0.027*	1.48	1.43–1.55	< 0.001*
VSR (high/low)	1.36	1.19–1.56	< 0.001*	1.42	1.36–1.48	< 0.001*
VAT density (high/low)	0.99	0.65–1.50	0.961	1.05	0.64–1.72	0.856
SAT density (high/low)	1.17	0.58–2.36	0.661	1.18	0.46–3.06	0.731

ALBI grade, albumin-bilirubin grade; AFP, alpha-fetoprotein; BCLC, Barcelona Clinic Liver Cancer; IMATI, intramuscular adipose tissue index; MASLD, metabolic dysfunction-associated steatotic liver disease; SATI, subcutaneous adipose tissue index; TKI, tyrosine kinase inhibitor; VATI, visceral adipose tissue index; VSR, VAT-SAT ratio

VATI cut-off value = 50 (high > 50; low <  = 50 [reference category]); SATI cut-off value = 48 (high > 48; low <  = 48 [reference category]); IMATI cut-off value = 5 (high > 5; low <  = 5 [reference category]), VSR cut-off value = 1 (high > 1; low <  = 1 [reference category]), SAT density cutoff value = -95 (high: > -95; low: <  = -95 [reference category]); VAT density cut-off value:-88 (high: > -88; low: <  = -88 [reference category])

*: Statistically significant

**Fig. 1:**
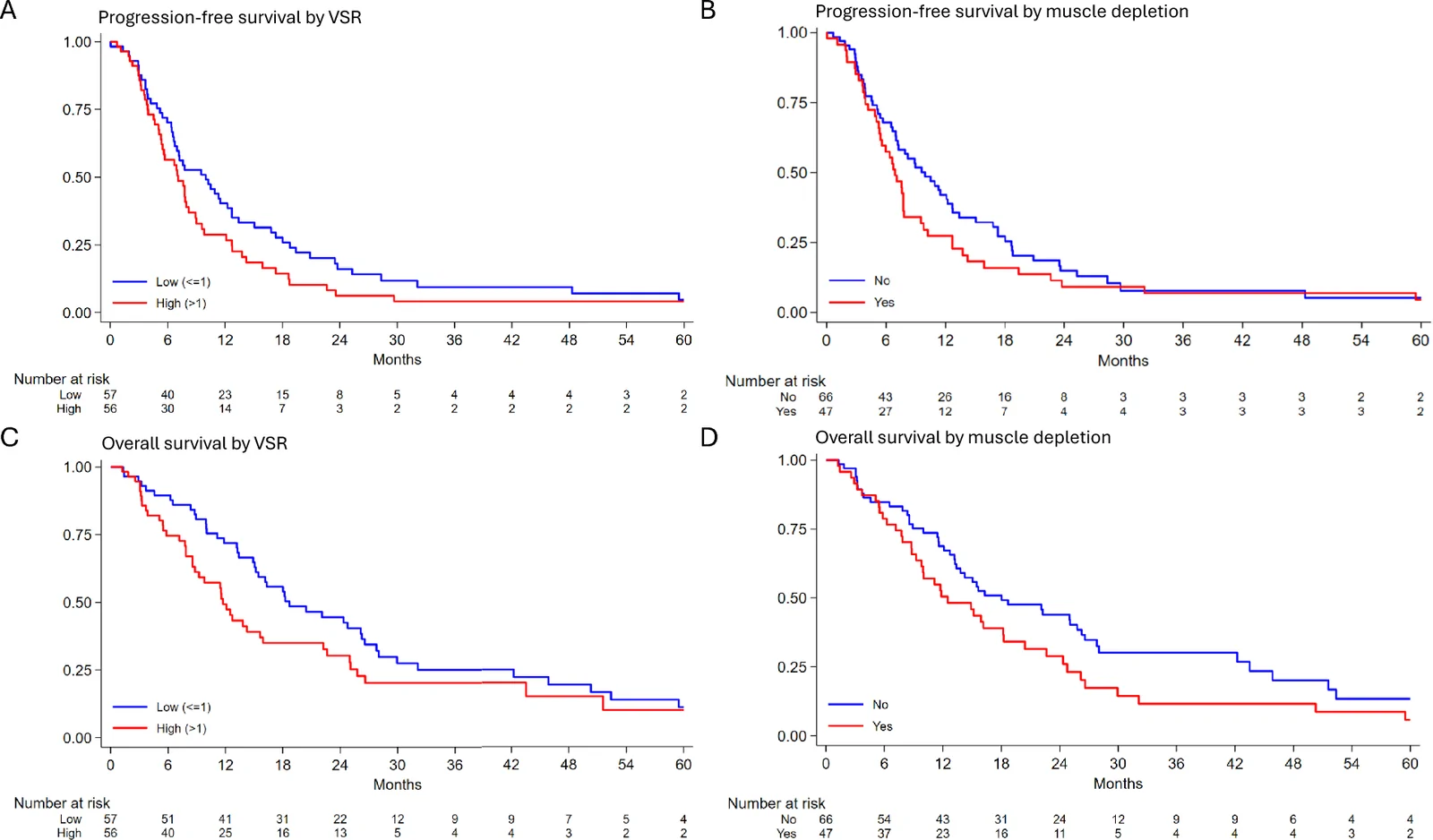
Kaplan–Meier survival curves for Progression-Free Survival (PFS) and Overall Survival (OS) according to visceral to subcutaneous adipose tissue ratio (below and above the median value of 1.0) at the level of the third lumbar vertebra (A and C), and according to muscle depletion (B and D)

Among BC parameters, muscle depletion, high VSR, and VATI were associated with worse PFS in the univariable analysis (HRs: 1.29, 1.36, and 1.26, respectively). However, only muscle mass and VSR retained their significant association with OS at univariable analysis, with poorer outcomes correlated with muscle depletion (HR: 1.48, 95%CI: 1.43–1.55, *p* < 0.001) and high VSR (HR: 1.42, 95%CI: 1.36–1.48, *p* < 0.001) (Fig. 1). VSR was shown to be a predictor of both PFS and OS in bivariable models adjusted for relevant prognostic factors (Supplementary Tables 2 and 3). An interaction between VSR and certain clinical features was observed; in these cases, subgroup analyses were performed (Supplementary Tables 4 and 5). All subgroup analyses showed a significant association between VSR and PFS, except in the subgroup with AFP < 400 ng/ml, in which VSR was not associated with PFS (Supplementary Table 4). Similar patterns were observed for OS (Supplementary Table 5). Notably, VSR was not associated with OS in cases with MASLD etiology, whereas an increased mortality was observed for other non-metabolic etiologies (HR 1.49, 95% CI 1.27–1.76).

### Body composition and dose-limiting toxicities

During the first-line treatment period, 75 patients (66.4%) experienced DLTs, resulting in dose reduction (n = 36; 48%), transient treatment suspension (n = 22; 29.3%), or treatment discontinuation (n = 38; 50.6%). The most commonly reported DLTs were fatigue (23.9%), arterial hypertension (18.6%), hand-foot syndrome, and diarrhea (13.3%) (Supplementary Table 6). The incidence of DLTs was lower in patients treated with ICI-based therapy than in those treated with TKI (HR 0.40; 95% CI: 0.38–0.43; *p* < 0.001) (Table 3). By contrast, macrovascular invasion, female sex, ALBI grade > 1, and AFP > 400 ng/ml were associated with a higher incidence of DLTs. Among baseline BC parameters, only VSR was significantly associated with a higher incidence of DLTs (HR 1.16; 95% CI 1.08–1.26; *p* < 0.001).

**Table 3:** Univariable Cox regression model for Dose-limiting toxicity in the overall population

Characteristics	HR	95% CI	*p* value
Gender (Female/Male)	1.33	1.02–1.73	0.038*
Age (≥ 70/ < 70 years)	1.12	0.71–1.74	0.628
AFP (> 400/ ≤ 400 ng/ml)	1.94	1.40–2.69	< 0.001*
Albi Grade (≥ 2/1)	1.63	1.54–1.73	< 0.001*
Child (B/A)	1.67	0.67–4.18	0.271
BCLC (C/B)	0.72	0.45–1.18	0.191
Macrovascular invasion (yes/no)	2.96	1.78–4.94	< 0.001*
Etiology (MASLD/Others)	1.48	0.71–3.07	0.295
Treatment regimen (Immunotherapy/TKI)	0.40	0.38–0.43	< 0.001*
Diabetes (Yes/no)	1.46	0.59–3.65	0.415
Body composition variables at baseline			
VATI (high/low)	0.97	0.53–1.79	0.924
SATI (high/low)	1.02	0.41–2.55	0.959
IMATI (high/low)	1.42	0.65–3.11	0.375
Muscle depletion (yes/no)	1.32	0.75–2.31	0.338
VSR (high/low)	1.16	1.08–1.26	< 0.001*
VAT density (high/low)	1.09	0.77–1.54	0.640
SAT density (high/low)	1.18	0.40–3.47	0.762

AFP, alpha-fetoprotein; ALBI grade, albumin-bilirubin grade; BCLC, Barcelona Clinic Liver Cancer; IMATI, intramuscular adipose tissue index; MASLD, metabolic dysfunction-associated steatotic liver disease; SATI, subcutaneous adipose tissue index; TKI, tyrosine kinase inhibitor; VATI, visceral adipose tissue index; VSR, VAT-SAT ratio. *: Statistically significant.

### Body composition dynamics

CT scan images at best response to first-line treatment were available for 85 patients (75.2%), and were obtained a median of 4.21 months (IQR: 3.12–5.99) after the baseline CT scan. Most of these patients (63.5%) were treated with the TKIs, specifically 41.2% with lenvatinib and 22.4% with sorafenib. Additionally, 31 patients (36.5%) received ICI-containing regimens, with 14 patients (16.5%) treated with atezolizumab-bevacizumab. Overall, 32 patients (37.6%) achieved stable disease as their best response, 4 patients (4.7%) achieved a complete response, and 26 patients (30.6%) achieved a partial response. Conversely, 23 patients (27.1%) experienced disease progression.

The mean changes in SMA, SAT, and VAT over time were − 5.01 (95%CI − 8.21 to − 1.81), − 18.89 (95%CI − 25.89 to − 11.90), and − 22.23 (95%CI − 34.22 to − 10.25), respectively, corresponding to a relative reduction of muscle mass, SAT, and VAT of 3.5%, 11.43%, and 13.04%, respectively (Table 4). In contrast, a significant increase in adipose tissue density was observed during treatment, with mean increases of 5.18% and 4.67% in VAT and SAT densities, respectively. Notably, no significant changes in VSR were observed over time.

**Table 4:** Body composition parameters at baseline and at the time of best response in patients with the best response CT scan available for analysis (N = 85)

Variables	Baseline CT scan Median (IQR)	Best response CT scan Median (IQR)	Mean change	95% CI	*p*- value
SMA, cm^2^	139.30 (125.35 to 162.55)	134.00 (115.65 to 152.75)	− 5.01	− 8.21 to − 1.81	0.003*
SAT, cm^2^	140.10 (104.50 to 222.20)	124.30 (88.72 to 193.30)	− 18.89	− 25.89 to − 11.90	< 0.001*
VAT, cm^2^	161.85 (63.37 to 250.70)	135.70 (61.98 to 205.80)	− 22.23	− 34.22 to − 10.25	< 0.001*
IMAT, cm^2^	15.47 (10.56 to 24.00)	13.41 (9.13 to 20.80)	− 2.56	− 4.68 to − 0.44	0.019*
VSR	0.96 (0.55 to 1.45)	0.92 (0.66 to 1.45)	0.01	− 0.07 to 0.09	0.774
VAT density, HU	− 90.04 (− 96.20 to − 81.17)	− 85.90 (− 92.73 to − 76.09)	5.18	1.05 to 9.32	0.015*
SAT density, HU	− 96.69 (− 103.80 to − 89.31)	− 93.39 (− 102.40 to − 82.77)	4.67	2.38 to 6.95	< 0.001*
SMI, cm^2^/m^2^	47.47 (43.02 to 56.19)	46.20 (40.16 to 52.97)	− 1.69	− 2.83 to − 0.56	0.004*
VATI, cm^2^/m^2^	57.81 (22.92 to 89.89)	48.89 (21.57 to 77.37)	− 7.85	− 12.14 to − 3.57	< 0.001*
SATI, cm^2^/m^2^	49.03 (36.18 to 71.46)	42.08 (30.50 to 62.67)	− 6.49	− 8.94 to − 4.03	< 0.001*
IMATI, cm^2^/m^2^	5.19 (3.65 to 8.00)	4.72 (3.12 to 6.77)	− 0.90	− 1.67 to − 0.12	0.024*

HU, Hounsfield unit; IMAT, intramuscular adipose tissue; IMATI, intramuscular adipose tissue index; SAT, subcutaneous adipose tissue; SATI, subcutaneous adipose tissue index; SMA, skeletal muscle area; SMI, skeletal muscle index; VAT, visceral adipose tissue; VATI, visceral adipose tissue index; VSR, VAT-SAT ratio. *: Statistically significant

No relevant differences in BC dynamics were observed between patients treated with TKIs and those receiving ICI-based therapies (no significant interaction). In bivariable models adjusted for treatment regimen, the temporal variation in BC parameters remains statistically significant (Supplementary Table 7). Muscle mass changes were positively correlated with VSR changes (R = 0.56, 95% CI 0.44–0.65), whereas no significant correlations were observed with SAT, VAT, or IMAT changes. (Supplementary Fig. 5).

Survival analysis according to BC at the time of best response revealed that high VSR, SAT density, and VAT density at T1 were associated with an increased risk of PFS. In contrast, only high VSR was associated with higher mortality (Table 5).

**Table 5:** Progression-free survival and Overall survival hazard ratios by muscle and adipose tissue subgroups at best response

Characteristics	Number of patients N = 85 (%)	PFS HR (95% CI)	*p* value	OS HR (95% CI)	*p* value
VATI at T1					
Low	50 (58.8)	1		1	
High or worsened	35 (41.2)	1.20 (0.77–1.86)	0.426	1.01 (0.35–2.95)	0.985
SATI at T1					
Low	51 (60)	1		1	
High	34 (40)	0.78 (0.52–1.17)	0.230	0.72 (0.23–2.22)	0.565
IMATI at T1					
Low	37 (43.5)	1		1	
High	48 (56.5)	1.11 (0.84–1.45)	0.457	1.21 (0.60–2.47)	0.595
VSR at T1					
Low	45 (53.0)	1		1	
High	40 (47.0)	1.37 (1.21–1.55)	< 0.001*	1.12 (1.09–1.16)	< 0.001*
Muscle Mass at T1					
Not depleted	38 (44.7)	1		1	
Depleted	47 (55.3)	1.39 (0.66–2.94)	0.388	1.72(0.62–4.75)	0.294
SAT density at T1					
Low	37 (43.4)	1		1	
High	48 (56.6)	1.39 (1.01–1.92)	0.040*	1.73 (0.54–5.53)	0.354
VAT density at T1					
Low	35 (41.2)	1		1	
High	50 (58.8)	1.29 (1.08–1.54)	0.006*	1.68 (0.92–3.07)	0.091

IMATI, intramuscular adipose tissue index; SATI, subcutaneous adipose tissue index; VATI, visceral adipose tissue index; VSR, VAT-SAT ratio

*: Statistically significant

## Discussion

In this study, we analyzed the impact of BC and its dynamics on survival and DLTs in a cohort of patients with advanced HCC undergoing first-line treatment with currently available therapies. Our findings indicate that muscle depletion and higher VSR values are associated with poorer outcomes in patients with HCC, suggesting that muscle mass and adipose tissue distribution are prognostic factors in this population. In particular, VSR confirms its relevant prognostic impact even after adjusting for treatment regimen and other significant risk factors, including liver function, tumor stage, and relevant comorbidities such as diabetes. These results align with previous research (Fujiwara et al. 2015; Montano-Loza et al. 2018; Hamaguchi et al. 2019; Shi et al. 2023). Recent evidence has shown that adipose tissue is not merely an energy-storage organ but a complex endocrine organ involved in various physiological activities that may contribute to tumor development and progression (Iyengar et al. 2003; Park et al. 2014; Blaszczak et al. 2021). Moreover, based on their anatomical location, adipocytes exhibit distinct metabolic and inflammatory profiles that are differentially associated with disease risk (Ramirez et al. 2020). In patients with cirrhosis, the balance between SAT and VAT becomes particularly important. Cirrhosis induces a chronic hypercatabolic state, prompting the body to utilize various metabolic pathways for energy production (Traub et al. 2021). At the adipose level, cirrhosis is characterized by increased lipolysis and reduced adipogenesis. In this context, SAT serves as a compensatory mechanism that counteracts energy depletion caused by heightened catabolism. The SAT not only secretes leptin and stimulates the insulin response but also activates carbohydrate and lipid metabolism, thereby contributing to the immune response through adipokine secretion (Iyengar et al. 2003). Conversely, VAT accumulation is linked to insulin resistance and leads to increased local and systemic inflammation, ultimately resulting in immune-senescence, particularly in elderly or sarcopenic patients (Lutz and Quinn 2012). These distinct metabolic and endocrine functions have led several studies to correlate reduced SAT and increased VAT volume with poorer prognosis in patients with cirrhosis and HCC, regardless of the disease’s stage and treatment received (Fujiwara et al. 2015; Montano-Loza et al. 2018; Hamaguchi et al. 2019; Imai et al. 2020; von Hessen et al. 2021; Zhu et al. 2023). A recent meta-analysis of 31 studies involving 7021 HCC patients found that, among the various adipose tissue distribution parameters analyzed, only VSR was significantly associated with survival (Shi et al. 2023). Specifically, consistent with our results, high VSR was associated with worse OS, with minimal heterogeneity across studies.

Subgroup analysis of OS showed that adipose tissue distribution had a particularly significant impact on prognosis among patients with non-metabolic underlying chronic liver disease. In contrast, no significant association with survival was observed among patients with MASLD, who were a subgroup underrepresented in our cohort (20.4% of the total). Adiposity plays a central mechanistic role in the pathogenesis of MASLD. Dysfunction of excess adipose tissue leads to increased free fatty acid flux, low-grade inflammation, and insulin resistance, all of which contribute to lipid accumulation in hepatocytes (Zhu et al. 2024). Research indicates that visceral adiposity, rather than overall obesity, is more strongly associated with MASLD and its progression to steatohepatitis and fibrosis (Cariou 2022). A possible explanation is that variability in adipose tissue distribution becomes less predictive of prognosis once HCC is established in this population. Patients with MASLD may share a relatively homogeneous metabolic–visceral adiposity phenotype, reducing the incremental prognostic value of VSR in this subgroup. However, given the small number of MASLD cases in our cohort, these findings should be interpreted with caution and confirmed in larger studies.

A non-significant correlation was observed between baseline low muscle mass and the incidence or type of DLTs. On the contrary, we observed a higher frequency of toxicities among patients with high values of VSR. The influence of adipose tissue on drug distribution and its crucial role in the metabolism of patients with cirrhosis and chronic liver disease may explain our findings. Visceral adiposity can substantially modulate drug pharmacokinetics, pharmacodynamics, and treatment tolerance. Mechanistically, visceral adiposity can contribute to an inflammatory environment by increasing the production of pro-inflammatory adipokines and altering immune-metabolic regulation, which—when combined with muscle wasting—may impair host immunity and treatment tolerance (Jang et al. 2021).

Analysis of changes in BC parameters during treatment showed that skeletal muscle mass, adipose tissue mass, and adipose tissue quality decreased over time in patients with HCC, consistent with previous reports (Imai et al. 2020). This occurred without significant differences among the various treatment regimens. In contrast, the overall distribution pattern of adipose tissue remained unchanged, as evidenced by the lack of variation in the VSR over time. This data suggests that the VSR, which accounts for differences in fat distribution, may serve as a negative prognostic factor that is minimally influenced by treatment and associated toxicities. However, the limited follow-up available and the fact that our analysis was restricted to BC changes during first-line treatment limit the generalizability of these results. Interestingly, no significant correlations were observed between muscle mass variations and SAT or VAT variations over time; on the contrary, a moderate positive correlation was shown between VSR and muscle mass variations (r = 0.56, 95% CI: 0.44–0.65), suggesting that in our population, muscle gains were accompanied by a more prominent increase of visceral relative to subcutaneous adipose tissue deposits. These findings suggest that the apparent improvement in prognosis expected from muscle gain may be counterbalanced by the adverse prognosis associated with an unfavorable VAT-to-SAT imbalance. If confirmed in larger, different cohorts, these trends merit further in-depth study to better understand the mechanisms underlying them.

Several limitations of our study should be acknowledged. First, the retrospective design, combined with a small sample size and the cross-sectional nature of our imaging-based BC assessment, restricts our ability to draw causal inferences or generalize our findings. Second, the best response was retrospectively assessed without formal RECIST. Given this limitation, the prognostic significance of BC parameters was evaluated not by their association with response outcomes but with survival. However, without standardized RECIST 1.1 or mRECIST analysis, the best response time point for body composition reassessment may be inconsistently defined across patients, thus partially limiting the generalizability of our results. Third, our analysis was limited to imaging-based assessments of muscle status and did not include critical muscle function parameters, such as handgrip strength, which are widely accepted as essential for diagnosing sarcopenia (Donini et al. 2022). The absence of data on muscle strength in our population precluded classifying patients with muscle depletion as sarcopenic. This may explain the lack of correlation observed between muscle mass and DLTs, in contrast to the results of recent studies that used bioelectrical impedance analysis (BIA) to assess muscle status (Cereda et al. 2024). Fourth, we did not monitor changes in body weight over time or assess food intake and physical activity levels, both of which may have substantial impacts on BC dynamics. Fifth, because recognized differences in adipose tissue quantity and quality exist across ethnicities and genders, the cutoff values for adipose tissue indexes derived from our findings cannot be generalized. The same applies to CT-based cutoffs for SMI (Martin et al. 2013), which were validated in a Canadian population of patients with lung and gastrointestinal cancer. Therefore, additional research with larger sample sizes is necessary to standardize adipose tissue parameters and validate our findings within a broader demographic context. However, based on the median values in our sample, the VSR cut-off in our study was set at 1. This allowed us to conclude that in our population, an imbalance in adipose tissue distribution—characterized by a predominance of visceral adiposity over subcutaneous adiposity—is associated with poorer outcomes and increased toxicity. This insight can facilitate further exploration in future studies. Moreover, although the absence of standardized, validated cut-off values for VSR in the literature may limit the reproducibility and clinical applicability of our results, a recent meta-analysis by Shi S et al. showed that VSR remained stable and had minimal heterogeneity despite slight variations in cut-off definitions across studies (Shi et al. 2023). The largest study to date on the prognostic significance of an adipose tissue BC parameters in HCC patients was published in the Journal of Hepatology by Fujiwara et al., who selected VSR cutoffs of 1.33 for men and 0.93 for women in a large retrospective cohort of 1257 patients with different stages of HCC (Fujiwara et al. 2015). Since two other studies later used the same cut-off values in their statistical analyses (Sawada et al. 2019; Okubo et al. 2021), we applied these cut-offs to our population. This unplanned exploratory analysis was consistent with the results obtained using the median values from our population: 34 patients (30.1%) were classified as having low VSR according to the Fujiwara cut-off, and high VSR according to this cut-off remained associated with a higher risk of progression (HR 1.36, 95% CI 1.35–1.38, *p* < 0.0001) and death (HR 1.39, 95% CI 1.18–1.63, *p* < 0.0001), suggesting the potential reproducibility of our results and strengthening the clinical utility of VSR as a prognostic factor (Supplementary Fig. 6).

Given the limited events-per-variable ratio, constructing a fully adjusted multivariable Cox model was not feasible without risking model overfitting and unstable estimates. Therefore, a structured series of bivariable Cox models and stratified subgroup analyses was utilized to assess the independent prognostic contribution of VSR relative to established clinical predictors. Although this approach does not fully replace formal multivariable adjustment, the consistent prognostic signal of VSR across analyses supports the robustness of these findings.

Including patients receiving diverse first-line treatment regimens may help mitigate the confounding effects of specific treatment toxicities on body composition dynamics. However, the small number of patients per treatment group limited our ability to assess the independent effect of body composition on each regimen. Addressing these limitations will be crucial for guiding future research and deepening understanding of these critical areas.

## Conclusions

Muscle mass and adipose tissue distribution are important prognostic indicators in HCC, but VSR emerged as a strong and stable predictor of survival and treatment toxicity. Future research should confirm these results in larger cohorts, clarify the molecular mechanisms linking adiposity distribution to treatment response, and assess their value for treatment stratification and clinical trial design.

## Supplementary Information

Below is the link to the electronic supplementary material.

Supplementary file1 (DOCX 5651 KB)

## Data Availability

The datasets generated and analyzed during the current study, along with information on the analytic methods used, are available from the corresponding author upon reasonable request.
