# Supplementary material for: Visceral-to-subcutaneous fat ratio is a time-stable predictor of survival and toxicity in advanced hepatocellular carcinoma
Source: J Cancer Res Clin Oncol. 2026 Aug 5;152(11):212. doi: 10.1007/s00432-026-06570-1 (PMC13731561; doi:10.1007/s00432-026-06570-1)
Supplement: Supplementary file 1 [file 432_2026_6570_MOESM1_ESM.docx]

**Supplementary Fig 1**

Inter-observer concordance of the CT measures of subcutaneous adipose tissue (A), Visceral adipose tissue (B), muscle mass (C), and intramuscular adipose tissue (D) in 20 randomly selected subjects. The concordance was excellent for all parameters, ranging from 0.983 to 1.

**Supplementary Fig 2**

Measurement of body composition parameters at the level of the third lumbar vertebra on a CT scan. Red: the total cross-sectional skeletal muscle area; green: intramuscular adipose tissue; yellow: visceral adipose tissue; blue: subcutaneous adipose tissue.

**Supplementary Figure 3**

STROBE flow diagram

1L, first-line; BCLC, Barcelona Clinic Liver Cancer

**Supplementary Table 1**

Patients’ anthropometric and body composition parameters.

| **Anthropometric variables** | **Overall (N=113)** | **Males (N=99)** | **Females (N=14)** | **p-value** |
| --- | --- | --- | --- | --- |
| Weight (kg),  Median (IQR) | 72  (67 to 85) | 74  (68.5 to 85.5) | 63  (55 to 66) | <0.001* |
| Height (m),  Median (IQR) | 1.7  (1.65 to 1.75) | 1.71  (1.66 to 1.75) | 1.60  (1.58 to 1.60) | <0.001* |
| BMI (kg/m^2^),  Median (IQR) | 25.35  (23.24 to 28.88) | 25.35  (23.45 to 29.04) | 23.71  (21.33 to 25.78) | 0.040* |
| **BMI categories** |  | | | |
| Underweight (BMI <18.5) | 2 (1.8) | 1 (1.0) | 1 (7.1) | 0.324 |
| Normal Weight (BMI:18.5-24.9) | 52 (46.0) | 44 (44.4) | 8 (57.1) |  |
| Overweight (BMI:25-29.9) | 37 (33.0) | 34 (34.3) | 3 (21.4) |  |
| Obese (BMI ≥30) | 22(19.5) | 20 (20.2) | 2 (14.3) |  |
| **Body composition parameters** |  | | | |
| SMA (cm^2^)  Median (IQR) | 139.3  (124.9 to 161.1) | 144.2  (130.7 to 162.8) | 90.55  (86.01 to 99.26) | <0.001* |
| VAT (cm^2^)  Median (IQR) | 160.3  (72.5 to 246.3) | 167.9  (75.7 to 253.8) | 83.7  (48.2 to 153.6) | 0.006 |
| SAT (cm^2^)  Median (IQR) | 142.3  (106.0 to 216.5) | 140.1  (104.5 to 216.5) | 172.3  (128.2 to 224.0) | 0.254 |
| IMAT (cm^2^)  Median (IQR) | 15.35  (10.56 to 23.38) | 14.55  (9.6 to 23.03) | 18.06  (15.35 to 25.91) | 0.042* |
| VSR  Median (IQR) | 0.95  (0.58 to 1.43) | 1.02  (0.61 to 1.46) | 0.46  (0.36 to 0.68) | <0.001* |
| SMI (cm^2^/m^2^)  Median (IQR) | 47.47  (43.02 to 55.10) | 48.88  (44.51 to 55.94) | 34.93  (33.60 to 38.02) | <0.001* |
| VATI (cm^2^/m^2^)  Median (IQR) | 53.95  (24.98 to 80.82) | 57.80  (25.02 to 90.47) | 34.95  (24.75 to 57.81) | 0.063 |
| SATI (cm^2^/m^2^)  Median (IQR) | 49.22  (36.18 to 71.46) | 48.70  (34.61 to 69.809 | 69.92  (50.08 to 84.31) | 0.043* |
| IMATI (cm^2^/m^2^)  Median (IQR) | 5.19  (3.65 to 8.08) | 4.74  (3.54 to 7.72) | 8.0  (6.0 to 10.12) | 0.008* |
| Muscle density (HU)  Median (IQR) | 37.78  (32.01 to 43.99) | 37.87  (32.01 to 44.02) | 33.93  (30.56 to 43.30) | 0.389 |
| VAT density (HU)  Median (IQR) | -88.22  (-94.83 to -79.85) | -89.23  (-95.32 to -80.81) | -85.64  (-94.83 to -79.31) | 0.502 |
| SAT density (HU)  Median (IQR) | -96.56  (-103.3 to -88.98) | -96.65  (-103.3 to -89.31) | -95.29  (-105.6 to -87.35) | 1.000 |
| IMAT density (HU)  Median (IQR) | -64.53  (-68.45 to -60.35) | -64.45  (-68.27 to -60.11) | -67.69  (-71.91 to -64.23) | 0.082 |

BMI, body mass index; HU: Hounsfield unit; IMAT, intramuscular adipose tissue; IMATI, intramuscular adipose tissue index; IQR: interquartile range; SAT, subcutaneous adipose tissue; SATI, subcutaneous adipose tissue index; SMA, skeletal muscle mass area; SMI, skeletal muscle index; VAT, visceral adipose tissue. VATI, visceral adipose tissue index, VSR: VAT-SAT ratio.

*: Statistically significant

**Supplementary Fig 4**

Correlations between body mass index and subcutaneous adipose tissue (A), visceral adipose tissue (B), skeletal muscle area (C), intramuscular adipose tissue (D), and the visceral-to-subcutaneous fat ratio (E). Blue circles represent individual patients

**Supplementary Table 2.**

Progression-free survival; bivariable Cox models

| **Variables** | **Categories** | **HR (95%CI)** | **p-value** | **Model p-value** | **p-value for interaction** |
| --- | --- | --- | --- | --- | --- |
| **Model: VSR and Age** | |  |  | 0.512 | 0.888 |
| VSR | Low | 1 |  |  |  |
|  | High | 1.38 (1.15-1.65) | 0.001* |  |  |
| Age | <70 years | 1 |  |  |  |
|  | ≥70 years | 1.24 (1.11-1.37) | <0.001* |  |  |
| **Model: VSR and ALBI grade** | |  |  | 0.841 | 0.154 |
| VSR | Low | 1 |  |  |  |
|  | High | 1.60 (1.17-2.19) | 0.003* |  |  |
| ALBI grade | 1 | 1 |  |  |  |
|  | ≥2 | 1.99 (1.87-2.12) | <0.001* |  |  |
| **Model: VSR and Child** | |  |  | 0.719 | 0.398 |
| VSR | Low | 1 |  |  |  |
|  | High | 1.53 (1.25-1.88) | <0.001* |  |  |
| Child | A | 1 |  |  |  |
|  | B | 2.02 (1.73-2.35) | <0.001* |  |  |
| **Model: VSR and BCLC** | |  |  | 0.162 | 0.576 |
| VSR | Low | 1 |  |  |  |
|  | High | 1.35 (1.17-1.57) | <0.001* |  |  |
| BCLC | B | 1 |  |  |  |
|  | C | 1.09 (0.96-1.23) | 0.186 |  |  |
| **Model: VSR and Macrovascular invasion** | |  |  | 0.165 | 0.494 |
| VSR | Low | 1 |  |  |  |
|  | High | 1.31 (0.85-2.00) | 0.218 |  |  |
| Macrovascular invasion | No | 1 |  |  |  |
|  | Yes | 3.01 (1.94-4.69) | <0.001* |  |  |
| **Model: VSR and Treatment regimen** | |  |  | 0.830 | 0.922 |
| VSR | Low | 1 |  |  |  |
|  | High | 1.38 (1.08-1.76) | 0.011* |  |  |
| Treatment regimen | TKI | 1 |  |  |  |
|  | Immunotherapy | 0.55 (0.39-0.77) | 0.001* |  |  |
| **Model: VSR and Diabetes** | |  |  | 0.334 | 0.292 |
| VSR | Low | 1 |  |  |  |
|  | High | 1.32 (1.19-1.45) | <0.001* |  |  |
| Diabetes | No | 1 |  |  |  |
|  | yes | 1.29 (0.70-2.37) | 0.415 |  |  |
| **Model: VSR and etiology** | |  |  | 0.666 | 0.167 |
| VSR | Low | 1 |  |  |  |
|  | High | 1.34 (1.21-1.48) | <0.001* |  |  |
| Etiology | Other | 1 |  |  |  |
|  | MASLD | 1.12 (0.76-1.66) | 0.555 |  |  |
| **Model: VSR and VATI** | |  |  | 0.846 | 0.874 |
| VSR | Low | 1 |  |  |  |
|  | High | 1.29 (0.99-1.67) | 0.059 |  |  |
| VATI | Low | 1 |  |  |  |
|  | High | 1.11 (0.80-1.54) | 0.543 |  |  |
| **Model: VSR and SATI** | |  |  | 0.835 | 0.879 |
| VSR | Low | 1 |  |  |  |
|  | High | 1.38 (1.22-1.56) | <0.001* |  |  |
| SATI | Low | 1 |  |  |  |
|  | High | 1.17 (0.91-1.509 | 0.241 |  |  |
| **Model: VSR and Muscle Depletion** | |  |  | 0.553 | 0.377 |
| VSR | Low | 1 |  |  |  |
|  | High | 1.38 (1.30-1.46) | <0.001* |  |  |
| Muscle depletion | No | 1 |  |  |  |
|  | Yes | 1.27 (1.03-1.57) | 0.024* |  |  |
| **Model: VSR and VAT density** | |  |  | 0.018 | 0.484 |
| VSR | Low | 1 |  |  |  |
|  | High | 1.38 (1.12-1.70) | 0.003 |  |  |
| VAT density | Low | 1 |  |  |  |
|  | High | 1.06 (0.72-1.57) | 0.760 |  |  |
| **Model: VSR and SAT density** | |  |  | 0.441 | 0.241 |
| VSR | Low | 1 |  |  |  |
|  | High | 1.45 (0.93-2.26) | 0.999 |  |  |
| SAT density | Low | 1 |  |  |  |
|  | High | 1.28 (0.51-3.23) | 0.596 |  |  |

ALBI grade, albumin-bilirubin grade; BCLC, Barcelona Clinic Liver Cancer; MASLD, metabolic dysfunction-associated steatotic liver disease; SATI, subcutaneous adipose tissue index; SMI, skeletal muscle index; TKI, tyrosine kinase inhibitors; VATI, visceral adipose tissue index; VSR, VAT-SAT ratio.

*: Statistically significant

**Supplementary Table 3**

Overall survival; bivariable Cox models

| **Variables** | **Categories** | **HR (95%CI)** | **P value** | **Model p-value** | **p-value for interaction** |
| --- | --- | --- | --- | --- | --- |
| **Model: VSR and Child** | |  |  | 0.508 | 0.497 |
| VSR | Low | 1 |  |  |  |
|  | High | 1.63 (1.55-1.71) | <0.001* |  |  |
| Child | A | 1 |  |  |  |
|  | B | 2.84 (1.45-5.54) | 0.002* |  |  |
| **Model: VSR and Macrovascular invasion** | |  |  | 0.456 | 0.953 |
| VSR | Low | 1 |  |  |  |
|  | High | 1.42 (1.11-1.81) | 0.005* |  |  |
| Macrovascular invasion | No | 1 |  |  |  |
|  | Yes | 2.55 (1.66-3.94) | <0.001* |  |  |
| **Model: VSR and Treatment regimen** | |  |  | 0.473 | 0.909 |
| VSR | Low | 1 |  |  |  |
|  | High | 1.46 (1.37-1.55) | <0.001* |  |  |
| Treatment regimen | TKI | 1 |  |  |  |
|  | Immunotherapy | 0.60 (0.41-0.86) | 0.006* |  |  |
| **Model: VSR and Diabetes** | |  |  | 0.141 | 0.926 |
| VSR | Low | 1 |  |  |  |
|  | High | 1.37 (1.33-1.42) | <0.001* |  |  |
| Diabetes | No | 1 |  |  |  |
|  | yes | 1.54 (0.79-3.02) | 0.202 |  |  |
| **Model: VSR and VATI** | |  |  | 0.253 | 0.524 |
| VSR | Low | 1 |  |  |  |
|  | High | 1.48 (1.14-1.94) | 0.004* |  |  |
| VATI | Low | 1 |  |  |  |
|  | High | 0.91 (0.48-1.71) | 0.766 |  |  |
| **Model: VSR and IMATI** | |  |  | 0.876 | 0.356 |
| VSR | Low | 1 |  |  |  |
|  | High | 1.43 (1.23-1.66) | <0.001* |  |  |
| IMATI | Low | 1 |  |  |  |
|  | High | 1.29 (0.73-2.27) | 0.383 |  |  |
| **Model: VSR and Muscle Depletion** | |  |  | 0.779 | 0.550 |
| VSR | Low | 1 |  |  |  |
|  | High | 1.47 (1.35-1.60) | <0.001* |  |  |
| Muscle depletion | No | 1 |  |  |  |
|  | Yes | 1.51 (1.49-1.52) | <0.001* |  |  |
| **Model: VSR and VAT density** | |  |  | 0.948 | 0.258 |
| VSR | Low | 1 |  |  |  |
|  | High | 1.46 (1.44-1.48) | <0.001* |  |  |
| VAT density | Low | 1 |  |  |  |
|  | High | 1.13 (0.75-1.72) | 0.560 |  |  |
| **Model: VSR and SAT density** | |  |  | <0.001* | 0.708 |
| VSR | Low | 1 |  |  |  |
|  | High | 1.52 (1.08-2.14) | 0.015* |  |  |
| SAT density | Low | 1 |  |  |  |
|  | High | 1.31 (0.40-4.31) | 0.658 |  |  |

IMATI, intramuscular adipose tissue index; IQR, interquartile range; SATI, subcutaneous adipose tissue index; TKI, tyrosine kinase inhibitors; VATI, visceral adipose tissue index; VSR, VAT-SAT ratio.

*: Statistically significant

**Supplementary Table 4**

Subgroup analysis of VSR hazard ratios for progression-free survival by subgroups with significant interaction test

| **Characteristic** | **Univariable model for PFS** | | | | |
| --- | --- | --- | --- | --- | --- |
|  | **PFS HR for VSR (high *vs* low)** | **95% CI** | **p-value** | **p-value for interaction** |  |
| **Sex** |  |  |  | <0.001* |  |
| Male | 1.48 | 1.40-1.57 | <0.001* |  |  |
| Female | 3.81 | 2.06-7.03 | <0.001* |  |  |
| **AFP levels** |  |  |  | <0.001* |  |
| <400 ng/ml | 1.16 | 0.91-1.48 | 0.234 |  |  |
| ≥400 ng/ml | 2.24 | 1.86-2.69 | <0.001* |  |  |
| **IMATI** |  |  |  | <0.001* |  |
| High | 1.26 | 1.23-1.28 | <0.001* |  |  |
| Low | 1.60 | 1.33-1.92 | <0.001* |  |  |

AFP, alpha-fetoprotein; IMATI, intramuscular adipose tissue index.

*: Statistically significant

**Supplementary Table 5**

Subgroup analysis of VSR hazard ratios for Overall Survival by subgroups with significant interaction test

| **Characteristic** | **Univariable model for OS** | | | | |
| --- | --- | --- | --- | --- | --- |
|  | **OS HR for VSR (high *vs* low)** | **95% CI** | **p-value** | **p-value for interaction** |  |
| **Age** |  |  |  | <0.001* |  |
| <70 years | 1.14 | 0.93-1.38 | 0.205 |  |  |
| ≥70 years | 1.78 | 1.68-1.87 | <0.001* |  |  |
| **Sex** |  |  |  | 0.017* |  |
| Male | 1.48 | 1.40-1.55 | <0.001* |  |  |
| Female | 5.98 | 5.37-6.66 | <0.001* |  |  |
| **AFP levels** |  |  |  | 0.004* |  |
| <400 ng/ml | 1.33 | 1.19-1.49 | <0.001* |  |  |
| ≥400 ng/ml | 1.78 | 1.55-2.05 | <0.001* |  |  |
| **ALBI grade** |  |  |  | <0.001* |  |
| 1 | 1.04 | 0.94-1.15 | 0.499 |  |  |
| ≥2 | 2.45 | 1.42-4.24 | 0.001* |  |  |
| **BCLC** |  |  |  | 0.010* |  |
| B | 1.43 | 1.40-1.46 | <0.001* |  |  |
| C | 1.52 | 1.39-1.66 | <0.001* |  |  |
| **SATI** |  |  |  | 0.001* |  |
| High | 1.08 | 0.95-1.23 | 0.263 |  |  |
| Low | 1.76 | 1.36-2.26 | <0.001* |  |  |
| **Etiology (MASLD/Others)** |  |  |  | 0.020* |  |
| MASLD | 1.03 | 0.83-1.28 | 0.785 |  |  |
| Others | 1.49 | 1.27-1.76 | <0.001* |  |  |

ALBI grade, albumin-bilirubin grade; AFP, alpha-fetoprotein; BCLC, Barcelona Clinic Liver Cancer; MASLD, metabolic dysfunction-associated steatotic liver disease; SATI, subcutaneous adipose tissue index.

*: Statistically significant

**Supplementary Table 6**

Dose-limiting toxicities in the overall population.

| **Adverse events type** | **Number of cases**  **N=113 (%)** | **Grade 1 or 2**  **N=113 (%)** | **Grade 3 or 4**  **N=113 (%)** |
| --- | --- | --- | --- |
| Fatigue | 27 (23.9) | 17 (15.0) | 10 (8.9) |
| Arterial Hypertension | 21 (18.6) | 16 (14.2) | 5 (4.4) |
| Hand-foot syndrome | 15 (13.3) | 8 (7.1) | 7 (6.2) |
| Diarrhea | 15 (13.3) | 10 (8.9) | 5 (4.4) |
| Liver decompensation | 13 (11.5) | 11 (9.7) | 2 (1.8) |
| Weight loss | 12 (10.6) | 9 (8.0) | 3 (2.6) |
| Immune-related toxicities | 10 (8.9) | 7 (6.2) | 3 (2.6) |
| Others | 28 (24.8) | 23 (20.3) | 5 (4.4) |

*According to the National Cancer Institute Common Terminology Criteria for Adverse Events, version 4.0

**Supplementary Table 7:** Bivariable models of body composition changes between baseline (T0) and best response (T1), and according to treatment regimen.

| **Bivariable models** | | **Mean difference** | **95% CI** | **p-value** | **Model p-value** |
| --- | --- | --- | --- | --- | --- |
| **Dependent variable** | **Independent variables** |  |  |  |  |
| **SMA** | T0 | 0 |  |  | 0.355 |
|  | T1 | -5.01 | -1.81 to -8.21 | 0.003* |  |
|  | TKIs | 0 |  |  |  |
|  | ICIs | -6.03 | -3.54 to -8.51 | 0.590 |  |
| **VAT** | T0 | 0 |  |  | <0.001* |
|  | T1 | -22.23 | -34.22 to -10.25 | <0.001* |  |
|  | TKIs | 0 |  |  |  |
|  | ICIs | -37.85 | -82.07 to 6.37 | 0.092 |  |
| **SAT** | T0 | 0 |  |  | <0.001* |
|  | T1 | -18.89 | -25.89 to -11.90 | <0.001* |  |
|  | TKIs | 0 |  |  |  |
|  | ICIs | -8.34 | -51.22 to 34.54 | 0.700 |  |
| **IMAT** | T0 | 0 |  |  | 0.054 |
|  | T1 | -2.56 | -4.68 to -0.44 | 0.019 |  |
|  | TKIs | 0 |  |  |  |
|  | ICIs | -0.98 | -5.61 to 3.66 | 0.677 |  |
| **VSR** | T0 | 0 |  |  | 0.540 |
|  | T1 | 0.01 | -0.07 to 0.09 | 0.775 |  |
|  | TKIs | 0 |  |  |  |
|  | ICIs | -0.18 | -0.50 to 0.15 | 0.276 |  |
| **VAT density** | T0 | 0 |  |  | 0.032* |
|  | T1 | 5.18 | 1.03 to 9.34 | 0.015* |  |
|  | TKIs | 0 |  |  |  |
|  | ICIs | 3.39 | -3.17 to 9.95 | 0.307 |  |
| **SAT density** | T0 | 0 |  |  | <0.001* |
|  | T1 | 4.67 | 2.38 to6.95 | <0.001* |  |
|  | TKIs | 0 |  |  |  |
|  | ICIs | 1.93 | -2.98 to 6.84 | 0.436 |  |
| **SMI** | T0 | 0 |  |  | 0.011* |
|  | T1 | -1.69 | -2.83 to -0.56 | 0004* |  |
|  | TKIs | 0 |  |  |  |
|  | ICIs | 1.57 | -2.58 to 5.72 | 0.454 |  |
| **VATI** | T0 | 0 |  |  | <0.001* |
|  | T1 | -7.85 | -12.15 to -3.56 | <0.001* |  |
|  | TKIs | 0 |  |  |  |
|  | ICIs | -14.15 | -29.52 to 1.21 | 0.071 |  |
| **SATI** | T0 | 0 |  |  | <0.001* |
|  | T1 | -6.49 | -8.95 to -4.02 | <0.001* |  |
|  | TKIs | 0 |  |  |  |
|  | ICIs | -3.72 | -18.56 to 11.11 | 0.619 |  |
| **IMATI** | T0 | 0 |  |  | 0.065 |
|  | T1 | -0.90 | -1.67 to -0.12 | 0.024* |  |
|  | TKIs | 0 |  |  |  |
|  | ICIs | -0.41 | -2.06 to 1.24 | 0.624 |  |

ICI, immune check-point inhibitors; IMAT, intramuscular adipose tissue; IMATI, intramuscular adipose tissue index; SAT, subcutaneous adipose tissue; SATI, subcutaneous adipose tissue index; SMA, Skeletal muscle area; SMI, skeletal muscle index; TKI, tyrosine kinase inhibitors; VAT, visceral adipose tissue; VATI, visceral adipose tissue index; VSR, VAT-SAT ratio.*:Statistically significant

**Supplementary Fig 5**

Correlations between changes in the skeletal muscle index and subcutaneous adipose tissue index (A), visceral adipose tissue index (B), intramuscular adipose tissue index (C), and the visceral-to-subcutaneous fat ratio (D) from baseline to the time of the best response to first-line treatment. Blue circles represent individual patients.

**Supplementary Figure 6:** Kaplan-Meier survival curves for Progression-Free Survival (A) and Overall Survival (B) by visceral-to-subcutaneous adipose tissue ratio, according to the cut-off values of 1.33 for men and 0.93 for women, previously used by Fujiwara et al. (J. Hepatol. 63 (1), 2015).

**A**

**B**
